# Towards human exploration of space: the THESEUS review series on cardiovascular, respiratory, and renal research priorities

**DOI:** 10.1038/npjmgrav.2016.31

**Published:** 2016-12-01

**Authors:** André E Aubert, Irina Larina, Iman Momken, Stéphane Blanc, Olivier White, G Kim Prisk, Dag Linnarsson

**Affiliations:** 1 Laboratory of Experimental Cardiology, Gasthuisberg University Hospital, KU Leuven, Leuven, Belgium; 2 Institute for Biomedical Problems, Moscow, Russia; 3 Université d’Evry Val d’Essonne, UBIAE (EA7362), Evry, France; 4 Université de Strasbourg, IPHC, Strasbourg, France; 5 CNRS, UMR7178, Strasbourg, France; 6 Université de Bourgogne, Dijon, France; 7 University of California, San Diego, CA, USA; 8 Karolinska Institutet, Stockholm, Sweden

## Introduction

The THESEUS project (Towards Human Exploration of Space: a EUropean Strategy) was initiated within the seventh Framework Programme by the European Commission. This project aimed to provide a cross-cutting, life science-based roadmap for Europe’s strategy towards human exploration of space, especially for deep space missions and its relevance to applications on Earth. To address these challenges, relevance of space research on the cardiovascular system, the lungs and kidneys, was discussed in an expert group and its principal conclusions will be presented in this article.

Human exploration of space has already a history of longer than 50 years (starting by the first flight of Yuri Gagarin on 12 April 1961). Since then, many short- and long-duration missions have taken place on a variety of platforms. It was soon found out that weightlessness affects almost all physiological systems such as muscular deconditioning,^1^ bone demineralization,^2^ cardiovascular deconditioning,^3^ alterations in immunology,^4^ cerebrovascular autoregulation,^5^ impaired cognitive processes and on nutrition and metabolism.^6,7^ These adaptive responses lead to a generalized deconditioning, more or less comparable to an accelerated aging process on Earth.^8^ However, luckily for the astronauts, this process is partially reversible on return to Earth. On the other hand, it might negatively affect the crew health and performance both in space and on return to Earth, i.e., orthostatic intolerance occurs frequently after spaceflight.^9^ In addition to these physiological factors, psychological factors (hostile environment, confinement…) elicit stress,^4,6^ influencing cardiovascular function.^10^

To circumvent the physiological drawbacks of spaceflight, many countermeasures have been implemented with mixed success so far.^3,11,12^ The most effective so far have been physical exercise, but with a cost price of being a very time-consuming occupation. Moreover, often the repetition of similar exercises is perceived as very boring by the astronauts with, as a consequence, a reluctance to perform it.^13^

With the exception of the Apollo Missions, all space flights have remained in the vicinity of Earth, the so-called ‘Low Earth Orbit’, and were limited in time. By remaining at this altitude, the influence of radiation was also limited.^14^ Only four astronauts spent more than a year in space in one run onboard the Russian MIR space station. The longest mission so far, lasting 437 days and 17 h, was conducted by Valery Polyakov (8 January 1994 until 22 March 1995).^15,16^ Since the arrival of Expedition 1 on 2 November 2000 of the International Space Station (ISS), the station has been continuously occupied, the longest continuous human presence in space. Until 2014, all missions to ISS have been limited to 6-month stay; contributing to the knowledge on the physiological changes associated with the adaptation of humans to short-term spaceflight.

To look for relevant threshold effects in health and performance, the ISS partners have decided to extend the stay of two crew members beyond the current 6-month stay to 12 months in 2015. This 1-year mission provides the opportunity to validate physical countermeasures applied to maintain bones, muscles and overall fitness, and use modern analysis techniques to identify any new areas of concern.

Human spaceflight is currently entering the next phase of space exploration, venturing towards the Moon, near Earth asteroids and Mars. Radiation levels will become more challenging, but this will be covered in a separate chapter. It is clear that such ambitious goals lead to inherent medical challenges. Therefore, the primary focuses of the next phase of bioastronautics research will be to further expand knowledge on the effects of long-duration spaceflight on the crew health and performance, to further develop efficient countermeasures and to facilitate post-flight re-adaptation to the terrestrial environment.

The THESEUS project aimed to provide a cross-cutting, life science-based roadmap for Europe’s strategy towards human exploration of space. This review, part of the cluster on integrated physiology, summarizes the findings of the expert group on cardiovascular physiology, including lungs and kidneys. These were considered as high priority disciplinary scientific topics or methodology issues representing challenges or opportunities for human space exploration, requiring further attention in the future and that may be relevant to the space community engaged in human spaceflight as well as to applications on Earth.

## What are the in-flight alterations in cardiovascular structure and function?

**Figure 1:**
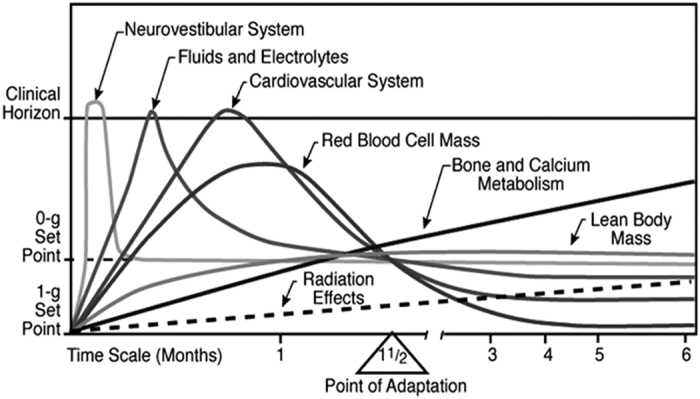
Changes of various body systems during adaptation to weightlessness^17^ (Credit: NASA (National Aeronautics and Space Administration)).

Some acute and chronic changes occur in the cardiovascular function upon becoming weightless (Figure 1).^6,17–22^ Figure 1 shows the initial acute effects, extending over a few days and some other effects extending over several weeks with a consecutive chronic decrease to a 0-g set point (with the use of an extensive countermeasures programme). Deleterious effects of bone deterioration and radiation will act linearly with duration of the space mission.

Cardiovascular adaptation:

* Impaired cardiovascular response to orthostatic stress.

* Diminished cardiac function.

* Impaired cardiovascular response to exercise stress.

**Figure 2:**
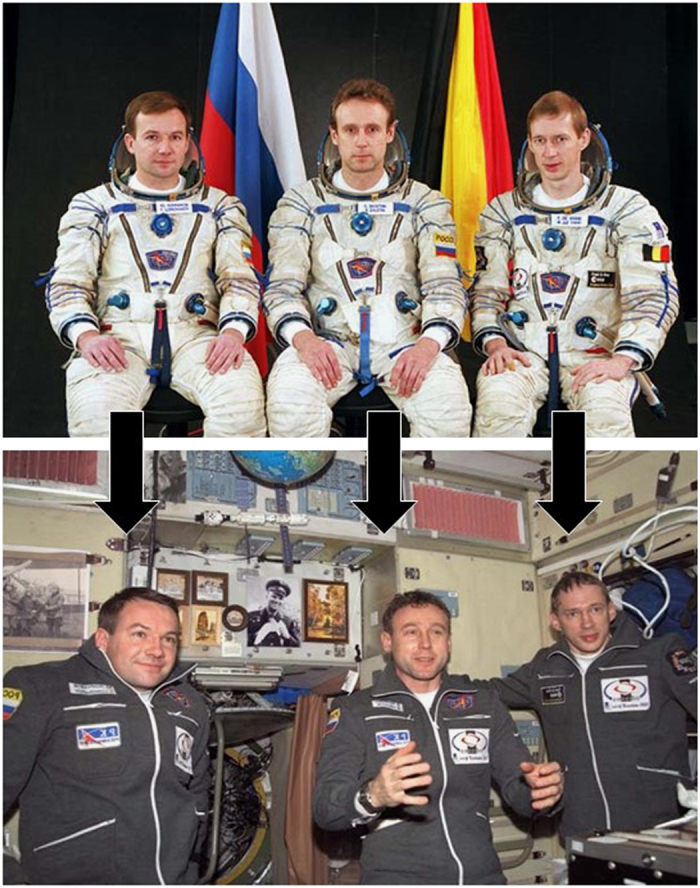
‘Puffy face’. Upper part: crew of Odissea Mission before launch, lower part: crew in-flight, first day (Credit: European Space Agency).

Among many other effects, weightlessness causes a substantial reduction in intrathoracic pressure by expansion of the thorax. The acute effects are a redistribution of blood and body fluids to the upper part of the body. This is the well-known effect of ‘puffy face’ and ‘chicken legs’ (Figure 2). Superficial tissue thickness in the tibia decreases by 15%, whereas it increases by 7% in the forehead.^23^

Understanding the dynamics of this fluid shift requires continuous monitoring of cardiac filling pressure: central venous pressure (CVP) measurement is the only way of obtaining this. Several groups were able to demonstrate a surprising decrease in CVP,^24,25^ together with increased cardiac chamber volumes. Weightlessness causes a substantial reduction in intrathoracic pressure by expansion of the thorax. The simultaneous cardiac expansion and CVP reduction during weightlessness were explained by Videbaek and Norsk^26^ by recording esophageal pressure (a measure of intrathoracic pressure) together with CVP and atrial diameter during parabolic flights. They found that the intrathoracic pressure drop was even larger than the decrease in CVP, such that cardiac transmural pressure increases in microgravity. Increased cardiac transmural pressure corresponded to increased atrial diameter. Also, the upward fluid shift will increase the blood volume in the trunk, which leads to atrial stretching due to the translocation of fluid in the headward direction. So it was clear that the mechanical consequences of entering microgravity lead to cardiac distension in the first 24 h of spaceflight. The atrial stretching will increase the amount of atrial natriuretic peptide^27^ and its secondary messenger cyclic guanosine monophosphate.^28^ An 80% increase of atrial natriuretic peptide was detected on the first day in microgravity,^29^ which will lead to an increased vascular permeability that together with the increased transmural pressure facilitates the transition of fluid and sodium from intravascular compartments to extravascular (intracellular, interstitial, lymphatic) spaces. The atrial natriuretic peptide will also induce vasodilatation.

Most recent findings reporting blood pressure show an adaptation when in space, with a set point corresponding to supine on the ground.^30–33^ This was also true for in-flight measurements of respiratory vagal-cardiac modulation and dynamic sympathetic vasomotor function. At the same time, stroke volume and cardiac output are increased and systemic vascular resistance decreased, whereas sympathetic nerve activity is kept surprisingly high. This would indicate that dilatation of the arteriolar resistance vessels by mechanisms other than a baroreflex induced decrease in sympathetic nervous activity.^31^

On one hand, this supports the hypothesis that weightlessness relaxes the circulation in humans for extended flight durations of upto 6 months.^33^ On the other hand, it poses the question to identify the mechanism inducing peripheral arteriolar dilatation (explaining behavior of blood pressure) and high sympathetic nerve activity and associated cardiovascular changes. Moreover a reduction in respiration frequency during prolonged microgravity exposure has been reported previously^34^ but is still difficult to interpret. An overall reduction in the total metabolic rate might provide an answer here;^35^ however, more studies will be needed, focusing on the cardiorespiratory coupling instead of examining these systems separately to provide definitive answers.

**Figure 3:**
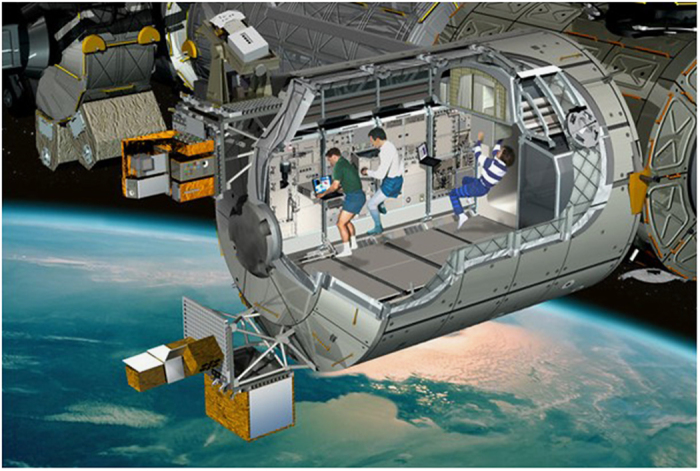
Columbus module of the ISS (Credit: European Space Agency).

Some answers could come from experiments being performed or planned on the Columbus module, that was attached to the ISS just 6 years ago (12 February 2008), especially from the European Physiology Modules Facility (Figure 3). The European Physiology Modules facility is designed to investigate the effects of long-duration spaceflight on the human body, with typical research areas including neuroscience, cardiovascular and respiratory system, bone and muscle physiology, and endocrinology and metabolism. The research into human physiology under weightless conditions will also contribute to an increased understanding of terrestrial problems such as the ageing process, osteoporosis, balance disorders, and muscle deconditioning. Cardiological problems can be investigated with the ‘Cardiolab’. This is a facility for investigating the different systems that are involved in the regulation of arterial blood pressure and the heart rate: ECG, Holter monitoring (ECG for 24 h or even longer) noninvasive blood pressure, noninvasive continuous blood pressure monitoring with finger cuff, respiration, impedance, echocardiography equipment and biomedical amplifiers. Data from Cardiolab will also be used to maintain the crew in good health during their stay on board, and to prepare the astronauts for their return to Earth.

### Earth benefits

Findings about blood pressure, fluid and electrolyte regulation in weightlessness are of relevance for understanding the mechanisms of some cardiovascular diseases. For example, heart failure patients exhibit salt and water retention, with the accumulation of fluid in the tissues (edema). The results from spaceflight investigations indicate that the heart is compressed in supine humans by gravity, which may cause further deterioration in the condition of heart patients. Investigations in space may reveal the degree to which gravity affects the failing heart and how to counteract it.^36^

By comparing the cardiovascular, hormonal, and kidney variables of healthy astronauts before flight, on Earth, and in space with heart patients, the following question might be answered: ‘How can astronauts, during prolonged spaceflight exhibit the same physiological patterns as heart failure patients, without being ill?’ An answer to this question might give insight into the pathophysiology of some heart diseases and lead to the development of new treatments.

### Brief review of latest developments

Cardiovascular function has been extensively studied over the last years by many groups from all over the world. From the European side, as described in the European Space Agency brochure: ‘Research and results from Columbus and the ISS’ (2012, pages 23): the first major programme in cardiopulmonary research on the ISS was the Cardiocog series of experiments that started during the Odissea mission with European Space Agency astronaut Frank De Winne in 2002 and was undertaken with numerous short- and long-term astronauts as test subjects.

Cardiocog was the foundation of European Space Agency’s cardiovascular research on the ISS and helped to increase the understanding of orthostatic intolerance.^30,32,33,37–42^

Some recent studies were on heart rate during sleep^43^ and related to physical activity in the ISS^44^ and baroreflex.^45–47^

Countermeasures used so far are mostly cornered around physical activity.^48,49^ The optimal countermeasure is yet to be defined but planned developments include artificial gravity^50^ and various exercise modes.^51^

### Knowledge gaps and research needs

The effects of prolonged spaceflight on myocardial mass,^52^ intrinsic contractility,^15^ myocardial compliance, and autonomic neuro-regulation^33^ are currently relatively unknown. Further, the interaction with peripheral circulation is complex and it is not known whether cardiac changes observed thus far are primarily cardiac or are due to secondary changes in the peripheral circulation. Again, results are expected from ongoing research coming from the Cardiolab rack in the Columbus module, especially from extra-long-duration space flights. Almost all necessary scientific equipment is present in the Cardiolab rack.

### Proposed investigations and recommendations

Long-duration missions present numerous risks to crew health and performance. These effects will be actively investigated leading to a better understanding of basic physiological processes to build a sustainable human space exploration programme for long-duration missions, binding for the next decades.^53^ Moreover, owing to the emerging field of commercial orbital and suborbital flights for civilian space travel, all risk factors should be soundly assessed.^54,55^ Although, the criteria applied to spaceflight participants are substantially less stringent than those for professional astronauts and/or crew members, inherent physiological risks related to spaceflight remain.

The following suggestions are made for further investigations and some remarks:^56,57^

- Dynamic responses of cardiac function to exercise/orthostatic tests simulating different phases of Moon and Mars exploration missions.^58,59^
- Bed rest simulations of relevant durations,^60^ and dry immersion simulations (up to 2 weeks).^61^
- Cardiac muscle structure by advanced echocardiography.^62^
- Simultaneous monitoring of (i) cardiac function (cardiac output), (ii) 24 h blood pressure, (iii) sympathetic nervous activity, (iv) plasma concentrations of vasoactive hormones (vasopressin, catecholamines, natriuretic peptides, and renin–angiotensin–aldosterone).^56,57^
- Monitoring of cardiovascular function during lower body negative pressure and dynamic exercise.^63^
- Develop tolerable 24 h blood pressure monitoring.^64^

As a justification for the more complex 24 h arterial blood pressure recording, as compared with single-point measurements, the latter are considered to be much less representative for the state of the subject and can be subject to emotional influences from the procedure itself (white coat hypertension).

- Detect the interactions between peripheral and central cardiovascular responses through the effects on conduit artery diameter, wall thickness and redistribution of venous blood flow by vessel imaging (ultrasound imaging)^58,65,66^ or MRI.

The following transdisciplinary aspects should also be considered:

- Thermoregulation changes in microgravity, but may impact both metabolic and cardiovascular control.^67^
- Radiation may interact with cardiovascular degradation/repair processes.^14^
- Mental stress and sleep disorders are likely to influence cardiovascular health.

## What is the influence of spaceflight on structure and function of blood vessels?

Orthostatic intolerance is frequently found after spaceflight^68^ and bed rest simulations,^69^ and may be a hazard to the astronaut and/or to other aspects of mission success including activities on the surfaces of Moon and Mars. The occurrence of orthostatic intolerance varies according to different sources: with a minimum of 0% in a study concerning 11 astronauts from missions to the ISS^33,70^ to 60% in a study from 14 astronauts on Shuttle missions (before the era of the ISS).^71^ It is to be noted, however, that the latter set of astronauts were on short-duration missions (10–14 days) with no countermeasures and the stand test was performed 4 h after landing.

Although this problem has not affected the success of any space mission so far, the concern is that this could impede crew members from performing an emergency evacuation in the event of a problem on landing. It is not certain that all crew members could evacuate a spacecraft without assistance immediately after landing. Although the orthostatic intolerance usually disappears quickly after spaceflight, the adaptation process after return to Earth can take several weeks.

### Earth benefits and applications

Orthostatic intolerance is frequently observed in patients after bed rest, during circumstances that reduce blood volume, and with the use of certain medications.^72^

In addition, blood pressure instability affects more than two-fifths of the population aged ⩾80 years, and may have a future role in the management of falls and syncope.^73^ More recently, orthostatic cerebral hypoperfusion syndrome, which is a novel syndrome of low orthostatic cerebral blood flow velocity, has been suggested a common cause of orthostatic dizziness.^74^

### Brief review of latest developments

Orthostatic intolerance has a multifactorial causation including reduced blood volume and attenuated constriction of resistance vessels. Orthostatic intolerance is present when an excessive postural decrease in cardiac filling and stroke volume and/or inadequate compensatory neurohumoral responses lead to failure to maintain adequate brain perfusion in the upright position. The loss of plasma volume has been attributed to directly trigger orthostatic intolerance; however, fluid volume restoring experiments with fludrocortisones did not alter the occurrence of presyncope,^75^ which indicates that the loss of plasma volume is not the primary mechanism for the orthostatic intolerance.

Blaber *et al*.^5^ demonstrated that astronauts who could not stand upright for 10 min upon return to normal gravity (nonfinishers) had a different cerebrovascular autoregulation than finishers. Thus, on the landing day, nonfinishers had a decrease of cerebrovascular conductance with standing, whereas the finishers did not.^5^ In contrast, Iwasaki *et al*.^76^ concluded that in-flight cerebrovascular regulation was preserved, if not even improved. In addition to these data from US Shuttle missions, cerebrovascular regulation has also been studied during more long-term ISS missions.^77^ These authors found impaired dynamic cerebrovascular autoregulation and reduced cerebrovascular CO2 reactivity. The pathophysiological significance of such findings with respect to orthostatic intolerance and impairments of visual acuity (see section on 'Vision Impairment and Intracranial Pressure' below) remain to be established and deserve further study.

Recent results implicate central-nervous processing and/or alpha adrenergic receptor dysfunction in post-flight orthostatic intolerance. There are pronounced gender differences^60^ as was clearly shown in the WISE study.^78^ For example women show an increased prevalence of orthostatic intolerance compared to men, and show greater losses of plasma volume following spaceflight than do men. This is accompanied by increased heart rate and vascular resistance.^79^ Whether or not this accounts for the greater tolerance to re-entry orthostasis is unclear and if countermeasures are to be used, a better understanding of the mechanisms involved is required.^79^

### Knowledge gaps and research needs

- The effects of long-term weightlessness on the structure of the arterial resistance vessels and to what degree it counteracts the development of hypertension is currently unknown.^80^
- It is currently unknown why the paradox exists that in space, sympathetic nervous activity is augmented, while the arterial resistance vessels are dilated. This has implications for understanding the role of gravity in the development of hypertension (see previous section).^81^
- Currently, little is known about the influence of space environment on the mechano-transduction signals and their influence on endothelial functions.^82^ However, the outcome of The Berlin Bed rest study showed that deconditioning is accompanied by a reduction in the diameter of the conduit arteries and by an increased reactivity to nitric oxide.^83^

### Proposed investigations and recommendations

- To simultaneously monitor cardiac output, blood pressure, sympathetic nervous activity, and plasma concentrations of vasoactive hormones (vasopressor and natriuretic vasodilatory substances), as well as the distribution of cardiac output to different vascular beds.
- Evaluate the effects of simulated low gravity on the structure and function of blood vessels, utilizing bed rest and other analogs such as dry immersion.

### Transdisciplinary aspects

The interaction between muscle and peripheral vessels should be considered in the design of experiments and countermeasures.

## What level of cardiovascular function loss is acceptable and what type and quantity of exercise is necessary that this loss is not exceeded?

### Relevance for space exploration missions

Countermeasures aimed at maintaining the full capacity of astronauts have a significant cost in terms of mass, energy, and time. However, it may not always be necessary to maintain this full capacity to complete a safe and successful space mission, and there may be an acceptable degree of deconditioning. It is hoped to learn a lot from the year-long missions to the ISS that was initiated during 2015. Compliance by the crew is also a major problem: None of the ISS crew members met the challenge of exercising twice a day without skip; in four cases, the dominating pattern was one session a day; in three cases, one session a day was made on half of the training days, and there was only one case when it was made in 90% of the training days.^13,84,85^

Not one countermeasure will be the answer to these problems, but a combination of different techniques will stand the most chance.

### Earth benefits and applications

Effective methods can also be used to prevent cardiovascular deconditioning or improve rehabilitation of patients on Earth. Also, more effective methods can be used on the ground to regain good health in sedentary populations and to maintain a good health status in aging populations.^8^ ‘Inactivity research’ is gaining more importance as humans are spending most of the time in sitting position. Deleterious effects include heart disease, diabetes due to increase of insulin resistance,^86^ colon cancer, muscle degeneration, circulatory disturbances, and low back pain.^87^

### Brief review of latest developments

**Figure 4:**
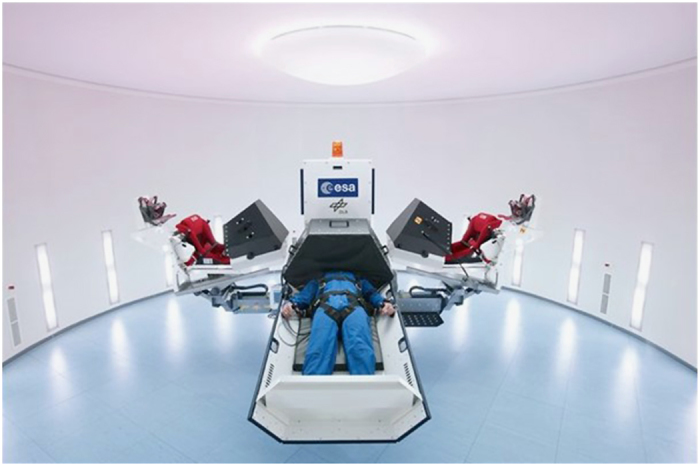
European Space Agency short-arm human centrifuge at DLR (Cologne). Maximum radius at outer perimeter: 2.8 m; maximum centrifugal acceleration: 4.5 g (foor level, test subject height: 1.85 m); number and type of nacelles: two beds, two seats; maximum overall payload: 550 kg (Credit ESA and DLR).

Neither resistive nor aerobic exercise alone is adequate to prevent deconditioning in spaceflight.^51^ The current exercise prescription used in ISS does not entirely prevent deconditioning. Much is hoped from application of artificial gravity (Figure 4) with centrifugation.^50^ However, this technology also needs to be evaluated carefully and submitted to a lot of research. Which gravity levels are sufficient? Does one need to reconstruct 1 g-force, or will partial g’s have the same effect? How long does the g-force need to be applied? Can a short-arm centrifuge, with its attendant head-to-foot variation in applied g-level adequately substitute for a long-arm centrifuge?

However, recently some drawbacks of this countermeasure have published: artificial gravity exposure impairs exercise related neurophysiological benefits.^88^ Little is known whether the transition form 0 G to 2–3 G and back to 0 G could elicit forms of travel or space sickness. So it must be carefully investigated to determine whether the cure is not worse than the disease!

### Knowledge gaps and research needs

- What is the requirement for cardiovascular performance on arrival at the Moon or Mars?
- What are the appropriate exercise prescriptions required to achieve such performance?
- How can the level of deconditioning that is acceptable to crews, who must be rehabilitated on return to Earth, be determined?

### Proposed investigations and recommendations

- On Earth, comparison studies of LBNP/exercise and centrifuge/exercise countermeasures focused on effectiveness on cardiovascular, muscular, bone, and neurovestibular systems are suggested.
- Different kinds and duration of exercises must be tested to determine the adequate amount of exercise needed. Short-term, middle-term, and long-term bed rest studies are required and gender studies.

### Transdisciplinary aspects

Countermeasures against cardiovascular degradation could/should be combined with countermeasures against musculoskeletal degradation. So far, this has mostly been achieved by cardiovascular physical exercise (equipment available on the ISS: CEVIS: Cycle Ergometer with Vibration Isolation System).^89–91^

## What are the mechanisms of the vision impairment and intracranial pressure syndrome and what are the ways to prevent it?

The vision impairment and intracranial pressure (VIIP) syndrome was first described in 2011.^92^ Many of the astronauts returning from space flights had developed an impaired visual acuity due to hyperopia (degradation in uncorrected near visual acuity). In a retrospective survey among astronauts, 23% of those returning from short-duration flights had experienced a worsening of their hyperopia. For those returning from long-duration flights, the corresponding value was 48%. In some individuals, these changes in visual acuity lasted for months or years after their return.

Ophthalmic findings and magnetic resonance images were obtained in seven astronauts before and after 6 months stay on the ISS. Findings were comparable as those typical of idiopathic intracranial hypertension, namely optic disk edema, globe flattening, choroidal folds, cotton wool spots, and distended sheets of the optical nerve. As a feasible mechanism for an elevated intracranial pressure in microgravity, the well-established fluid and blood shifts in cranial direction has been suggested.^93^ Other factors, which may promote an elevation of intracranial pressure include elevated carbon dioxide levels in the cabin air, and increases in intrathoracic pressure during resistive training.

In contrast to the ophthalmic findings, the subjective symptoms of the astronauts differed from idiopathic intracranial hypertension; none of the astronauts presented with chronic headaches, transient visual obscurations, or pulse-synchronous tinnitus, of which all are symptoms typical of idiopathic intracranial hypertension, and that speaks against elevated intracranial pressure as the sole mechanism for the ophthalmic findings.^94^ For practical and ethical reasons, actual invasive measurements of intracranial pressure have not been possible. Indirect data obtained post-flight by lumbar puncture in a few astronauts have not been conclusive.

Alternative mechanisms for VIIP include a concomitant lowering of the intraocular pressure and/or local changes in the fluid exchange between the space around the optic nerve and the surrounding tissue.^94^ Nevertheless, elevated intracranial pressure remains as a likely important factor^93^ and hence the term VIIP has been generally accepted.

### Relevance for space exploration missions

The perspective that astronauts may suffer from a chronic elevation of the intracranial pressure during long-term exploration space mission is indeed frightening; so if the VIIP syndrome could not be understood and prevented, that would be an obstacle for such missions. The concern of the human spaceflight community is reflected by a recent, intense research activity (see 'Brief review of latest developments' below).

### Earth benefits and applications

Elevation of the intracranial pressure occurs in many serious clinical disorders and efforts to develop, improve, and test noninvasive monitoring techniques for use in space will be to the benefit of many patients with such disorders.

### Brief review of latest developments

The VIIP syndrome has been defined only recently, so the introductory text above summarizes the recent developments as reflected in the published literature. An inventory of the research project catalogs of NASA (National Aeronautics and Space Administration) and other national space agencies show that some 30 research projects related to VIIP have been initiated since 2011 (status as of January 2016). Projects include monitoring of ophthalmic structures before, during, and after spaceflight, animal models of VIIP, studies of the fluid distribution in the cranial parts of the human body, interventions to reduce central venous pressure, and noninvasive estimation of intracranial pressure. Changes in intracranial pressure can be estimated from changes in tympanic membrane displacement,^95^ a method that possibly can be developed further.

### Knowledge gaps and research needs

Although the changes in ophthalmic structures can be monitored noninvasively in-flight, this is not true for absolute values of intracranial pressure. Therefore, it remains to be shown whether a rise in intracranial pressure really is a parallel phenomenon to the well-documented changes of vision and the geometry of the eye and related structures. So far, there are no established methods to mitigate or prevent the VIIP syndrome.

### Proposed investigations and recommendations

- Studies of animal models of VIIP on earth.
- Development of improved noninvasive methods to monitor intracranial pressure.
- Analysis of environmental factors on ISS (e.g., oxygen and carbon dioxide in the breathing air, training regimens) and whether such factors could be related to VIIP.
- Studies of cerebrovascular autoregulation in astronauts with and without VIIP
- The cerebrovascular response to carbon dioxide (CO_2_) in post spaceflight as a consequence of elevated partial pressure of CO_2_ in the ambient air such as in ISS could chronically influence arterial partial pressure of CO_2_ (PaCO_2_) and arterial acid–base balance that affect cerebral cerebrovascular resistance.^77^

### Transdisciplinary aspects

A truly cross-disciplinary approach is necessary here including experts in cerebrovascular dynamics, ophthalmology, neuroscience, and aerospace medicine.

## What are the risks associated with exposure to extraterrestrial dust?

Also the behavior of the pulmonary system during weightlessness, has been extensively studied over the last 50 years of manned spaceflight.^96,97^ Despite the changes in lung function, however, when gravity is removed, the lung continues to function well during weightlessness. Unlike many other organ systems, the lung does not appear to undergo structural adaptive changes during weightlessness, and so there is no apparent degradation in the lung function on return to Earth, even after 6 months in space.^98^

The only direct human exposure to extraterrestrial dust so far has been during the Apollo missions. On the other hand, dust accumulation on the surfaces of critical instruments has been a major concern during lunar and Mars missions. The operation of instruments such as solar panels, chromatic calibration targets … have been severely compromised in the past as a result of dust accumulation and adhesion. Wind storms on Mars, with wind speeds of upto 110 km/h (with the occurrence of so-called ‘Dust Devils’, i.e., miniature tornados) have not been effective in removing significant amounts of the deposited dust. This is indeed an indication of the strength of the adhesion force(s) involved between the dust particles and the surface(s) that they have adhered to.^99^

Lunar and Martian dust may be a toxic challenge to astronauts. Although deposition in reduced gravity is less than in normal gravity (1 G), reduced gravitational sedimentation causes particles to penetrate deeper in the lung, potentially causing more harm.^100^

**Figure 5:**
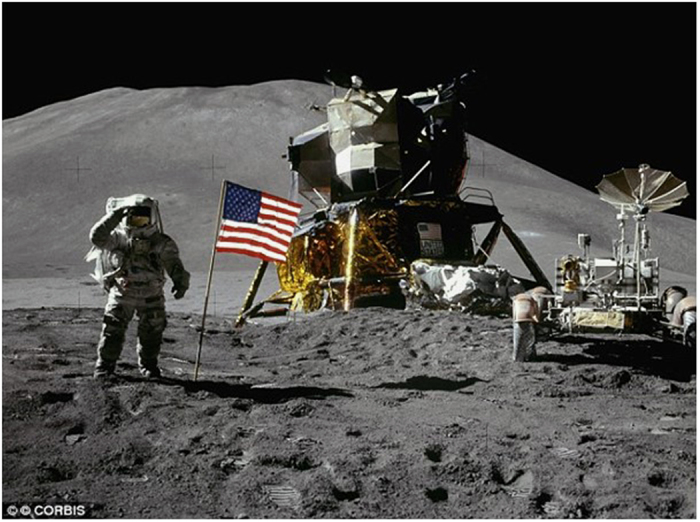
Lunar dust stuck to the astronaut’s spacesuit will inevitably be transferred into the space habitat (Credit: NASA (National Aeronautics and Space Administration)).

**Figure 6:**
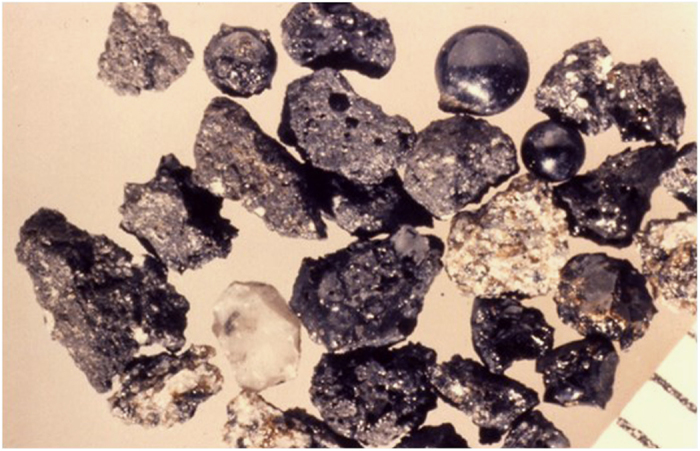
Lunar dust is covered in a glassy coating that can either be smooth or jagged (Credit: Larry Taylor).

In exploration class missions, crews will be exposed to extraterrestrial dusts that will inevitably be transported into the habitats (Figure 5), and these dusts may potentially be highly toxic.^100^ These fine dusts can easily be inhaled, and in a low-gravity environment, the particles will be transported deeper into the lung where they will likely remain for extended periods of time. This will likely enhance their toxicity (Figure 6). These problems have to be carefully addressed for developing appropriate habitats and space suits for the Moon and Mars.

### Earth benefits and applications

Many workers are exposed to dusty work environments, and particulate matter in the environment is a known health risk to urban populations. Acute exposure to air pollution has also been linked to cardiovascular diseases.^101^

Further, many drugs are now delivered in aerosol form, and therefore a comprehensive understanding of the deposition and subsequent clearance of deposited particles is of considerable importance in both the areas.

### Brief review of latest developments

The European Space Agency has plans for unmanned missions to define the physical and chemical properties of lunar dust in the South Polar Region.

### Knowledge gaps and research needs

- Size distribution, toxicity and physical properties of the dust
- The spatial pattern of particle deposition in micro and low gravity is unknown.
- Lung clearance rates in micro and low gravity are unknown. Past and ongoing studies suppose clearance rates in low gravity are identical to 1 G.
- Individual variation in airway tree geometry might be a major determinant of particle deposition and subsequent clearance.

### Proposed investigations and recommendations

- Studies of toxicity of lunar dust and its passivation rate performed either *in situ* (would require unmanned lunar surface exploration) or on Earth using samples collected from future lunar missions and stored in appropriate condition to maintain their ‘freshly fractured’ properties.
- Study the effects of micro and low gravity on the clearance rate. This will require access to human suborbital or orbital flights.
- Scintigraphic and/or fluorescent imaging studies of aerosol deposition and subsequent clearance in the lung either in humans or in an animal model with a focus on the alterations in both deposition and clearance that result from reduced gravity.

### Transdisciplinary aspects

Radiation may interact with pulmonary degradation/repair processes.

## What are the roles of diet and bone demineralization on kidney stone formation and is it possible to predict the risk of kidney stones?

The most obvious changes observed in astronauts are the increased excretion of calcium and the negative calcium balance thought to be the result of bone loss. Negative calcium balance was observed during Skylab and MIR missions. Increased urinary and fecal calcium excretion accounted for most of the deficit.^102,103^ Increased urinary calcium excretion is a major contributor to the increased risk of renal stone formation during and after spaceflight.^104^ Other demonstrated changes include decreased urinary output, urinary pH, magnesium, and citrate concentrations and increased urinary phosphate. These changes are expected to increase the urinary supersaturation of renal stone-forming salts and increase the risk of renal stone formation. In addition, to overcome the volume regulatory alteration such as renal renin–angiotensin–aldosterone system, cardioendocrine, and cardiovascular system alterations, the suggestion was made to apply high-salt diet,^105^ however, this could be a contributor to kidney stone formation.

### Relevance for space exploration missions

Despite the fact that astronauts are pre-screened during selection to be non-kidney stone formers, a significant number of events with renal stones have been reported from the US and Russian space programs.^106,107^ A calculus in the urinary system could have significant health consequences for an astronaut and could also have a negative impact on the mission as a whole.

### Earth benefits and applications

A better insight into the factors that cause an increased risk for renal stone formation in astronauts is likely to benefit many individuals on the ground that suffer from renal stones or an increased likelihood of suffering from renal stones.^108^ Apart from being extremely painful, renal stones may also lead to complications such as infections and hydronephrosis.

### Brief review of latest developments

Ingestion of sufficient amounts of fluids and of potassium citrate in astronauts^104^ has been shown to counteract changes in urinary composition that are known to favor renal stone formation. Other demonstrated changes include decreased urinary output, urinary pH, magnesium, and citrate concentrations, and increased urinary phosphate. These changes are expected to increase the urinary supersaturation of renal stone-forming salts and increase the risk of renal stone formation.

### Knowledge gaps and research needs

- Can regimes other than fluid and potassium citrate ingestion mitigate renal stone formation?
- Can potassium citrate ingestion increase the risk for brushite stones?
- How does renal stone formation relate to bone loss during very long space missions?

### Proposed investigations and recommendations

- Further studies should be performed of the mechanisms for renal stone formation during the post-flight period.
- Improved methods to identify the risks for renal stones at the time of selection should be developed, as present routines have proven inadequate.
- Improved methods to suppress bone atrophy should be developed.

### Transdisciplinary aspects

The interactions between kidney stone formation and nutrition must be considered, and a close coordination with programs to prevent bone atrophy appears necessary.

## Conclusion

After 50 years of spaceflight, we are beginning to understand some mechanisms of physiological adaptations; however, new questions develop all the time because of sometimes unpredicted results. There is still a long way to go before we can safely send humans to Mars and bring them back safely. The very long-duration missions to the ISS (1 year or even longer) will provide a unique opportunity to study the long-term effects of microgravity on the human body, extending the knowledge base from the Russian long-duration experience.

The importance of these physiological adaptations also has to be assessed in relation to the duration of the space mission. Exploration missions into deep space, such as a journey to Mars or to asteroids, may raise a series of new questions about the health of the human participants. Some physiological effects of shorter duration, such as bone mineral density decrease, are likely to continue indefinitely during longer missions. Some risks may be increased, such as radiation exposure. Also psychological and mental health issues will grow increasingly important during long-duration missions. The high noise levels, less than optimal light conditions, and confinement to small living quarters will be contributors to high psychological and social stress levels that are also non-negligible factors, certainly in long-duration space flights.

To gain support of the general public for manned spaceflight, it is also important that it receives information about the Earth-bound applications of space research. The development of diagnostic tools and of therapies for osteoporosis, syncope, heart failure, and muscle deconditioning will enhance public support and political will to continue and increase the intensity of human space exploration.
